# Discordance Between Clinical Suspicion and Spirometry-Defined Reversible Airflow Obstruction in Children with Sickle Cell Disease in French Guiana

**DOI:** 10.3390/jcm15166342

**Published:** 2026-08-17

**Authors:** Gabriel Bafunyembaka, Estiverne Evens, Nadia Nathan, Arriel Makembi, Narcisse Elenga

**Affiliations:** 1 Department of Pediatrics, University Hospital of Guiana, Saint-Laurent-du-Maroni Site, 1465 Boulevard de la Liberté, Saint-Laurent-du-Maroni 97320, French Guiana; e.estiverne@chu-guyane.fr; 2 Department of Pediatrics, General Reference Hospital of Bukavu, Avenue Michombero, Bukavu, South Kivu, Democratic Republic of the Congo; 3 Pediatric Pulmonology Department, Armand Trousseau Hospital, Assistance Publique—Hôpitaux de Paris, Sorbonne Université, 26 Avenue du Dr Arnold Netter, 75012 Paris, France; nadia.nathan@aphp.fr; 4 Department of Internal Medicine, University Hospital of Guiana, Saint-Laurent-du-Maroni Site, 1465 Boulevard de la Liberté, Saint-Laurent-du-Maroni 97320, French Guiana; a.makembi@chu-guyane.fr; 5 Department of Pediatrics, Cayenne Hospital, 3 Avenue Alexis Blaise, Cayenne 97300, French Guiana; elengafr@yahoo.fr

**Keywords:** sickle cell disease, asthma, clinical suspicion, spirometry, reversible airflow obstruction, bronchodilator reversibility, children

## Abstract

**Background**: Asthma is an important respiratory comorbidity in children with sickle cell disease (SCD), but respiratory symptoms overlap with SCD-related pulmonary manifestations. **Methods**: We analyzed prospectively recorded routine-care data from 140 children aged 5–17 years who were offered a standardized respiratory assessment during clinically stable follow-up. During revision, we applied a post hoc operational spirometric comparator: reversible airflow obstruction required FEV_1_/FVC < 80% plus an FEV_1_ increase ≥12% after salbutamol. Participants with normal spirometry formed the negative comparator; incomplete or intermediate objective patterns remained unclassified. **Results**: Reversible obstruction was identified in 43 children, normal spirometry in 25, and 72 remained unclassified. Previous clinical suspicion was present in 10/43 (23.3%), 3/25 (12.0%), and 22/72 (30.6%), respectively. In the deliberately contrastive classified-only analysis (*n* = 68), sensitivity was 23.3% (95% CI 11.8–38.6), specificity 88.0% (68.8–97.5), LR+ 1.94 (0.59–6.39), LR− 0.87 (0.70–1.09), and κ = 0.090 (95% CI −0.059 to 0.239). The likelihood-ratio intervals were wide and included 1.0, indicating substantial imprecision rather than proof of no discrimination. **Conclusions**: Prior clinical suspicion showed low sensitivity and slight agreement with spirometry-defined reversible obstruction. Interpretation is limited by the post hoc two-gate comparison and by the large, non-randomly distributed unclassified group.

## 1. Introduction

Sickle cell disease (SCD) is the most common severe inherited hemoglobin disorder worldwide and remains associated with substantial morbidity, premature mortality, recurrent healthcare use, and progressive multiorgan damage. Its global burden has continued to increase, with particularly marked consequences in populations of African ancestry and in regions where access to comprehensive care remains unequal [1,2]. Although vaso-occlusive crises, anemia, infection, stroke, and acute chest syndrome are among its most recognized complications, chronic and recurrent respiratory disorders also contribute substantially to disease burden in children with SCD.

Pulmonary involvement may begin early in life and can manifest as recurrent wheezing, cough, exercise intolerance, nocturnal symptoms, hypoxemia, airway obstruction, sleep-disordered breathing, or episodes of acute chest syndrome. Respiratory infections, ventilation–perfusion abnormalities, inflammation, endothelial dysfunction, and pulmonary vascular injury may interact with hemolysis and vaso-occlusion, making respiratory symptoms both clinically important and difficult to interpret [3,4]. Early recognition of treatable respiratory comorbidities is therefore considered an important component of pediatric SCD care.

Asthma is one of the most clinically relevant respiratory comorbidities in children with SCD. Its coexistence has been associated with increased pulmonary morbidity, particularly acute chest syndrome. A recent systematic review and meta-analysis found that patients with both SCD and asthma had a substantially greater risk of acute chest syndrome than those without asthma [4]. In French Guiana, asthma has also been associated with a greater burden of acute vaso-occlusive morbidity among children with SCD [5]. These observations support active identification and appropriate treatment of asthma in this high-risk population.

However, diagnosing asthma in children with SCD is particularly challenging. Symptoms commonly used to recognize asthma—wheezing, cough, dyspnea, chest discomfort, exercise limitation, and nocturnal respiratory symptoms—may also result from anemia, previous acute chest syndrome, recurrent infection, deconditioning, chronic airway inflammation, or SCD-related pulmonary injury. Conversely, children may have clinically important airflow limitation or bronchodilator responsiveness without a previously documented diagnosis of asthma. Clinical recognition may therefore be affected by both underdiagnosis and misclassification.

The diagnosis of childhood asthma is itself imperfect, even outside the context of SCD. No individual symptom, questionnaire, biomarker, or pulmonary function test provides an absolutediagnostic reference standard [6,7].Current European Respiratory Society recommendations emphasize that asthma should not be diagnosed solely from clinical history or from a single abnormal test. In children aged 5–16 years, objective evaluation using spirometry, bronchodilator reversibility testing, and, where available, fractional exhaled nitric oxide is recommended to support diagnostic confirmation [8]. Nevertheless, objective testing remains inconsistently performed, and differences between guidelines regarding test selection and diagnostic thresholds contribute to variation in clinical practice [6,9].

Clinical assessment alone may be especially unreliable because respiratory symptoms are episodic, caregiver reporting can be imprecise, and physical examination is frequently normal between exacerbations. Studies comparing parental reports, physician diagnosis, and spirometry have demonstrated limited agreement between these approaches [7]. Thus, a previous medical diagnosis may not always be objectively confirmed, while children without a documented diagnosis may demonstrate reversible airflow obstruction when systematically tested.

Spirometry is the most widely recommended objective test for children old enough to perform reproducible respiratory maneuvers. Measurement of the forced expiratory volume in one second, forced vital capacity, and their ratio allows identification of expiratory airflow limitation, while post-bronchodilator testing assesses reversibility. However, normal baseline spirometry does not exclude asthma because airway obstruction may vary over time, particularly in children with intermittent symptoms [9]. Conversely, abnormal spirometry in SCD may not necessarily indicate conventional asthma, because obstructive, restrictive, mixed, and unclassified pulmonary abnormalities can arise through disease-specific mechanisms. Objective results must therefore be interpreted alongside symptom patterns, previous respiratory events, treatment response, andclinical context [10,11,12].

Questionnaires may facilitate systematic detection of respiratory symptoms when specialist assessment and pulmonary function testing are not routinely available. Instruments derived from the International Study of Asthma and Allergies in Childhood have been widely used to estimate wheezing and asthma-related symptom burden in epidemiological studies. However, symptom-based questionnaires were primarily designed for population surveillance and do not independently establish a clinical diagnosis. In children with SCD, their performance may be further affected by overlap between asthma symptoms and disease-related respiratory manifestations. A positive questionnaire may therefore increase clinical suspicion but still requires standardized study assessment.

Previous screening studies in pediatric SCD have shown that structured questionnaires and pulmonary function testing may identify children with previously unrecognizedrespiratory abnormalities [13,14]. Pulmonary subspecialty care has also been associated with reduced subsequent acute respiratory healthcare use in children with SCD and asthma [15].Questionnaire findings, clinical labels, and objective testing do not necessarily identify the same children, a discordance also reported in general pediatric asthma assessment [7]. More recent phenotyping work in SCD has shown that spirometric indices, airway resistance, exhaled nitric oxide, and inflammatory or allergic markers may provide complementary rather than interchangeable information [16]. These findings support objective respiratory assessment while underscoring that no single spirometric pattern is synonymous with a definitive asthma diagnosis.

The consequences of missed clinically relevant airway disease may be particularly important in SCD. Unrecognized or undertreated airway disease may contribute to recurrent respiratory symptoms, exercise limitation, nocturnal hypoxemia, emergency visits, and progression of respiratory infections or vaso-occlusive events toward acute chest syndrome. Conversely, inaccurate labeling of asthma may expose children to unnecessary long-term treatment while diverting attention from other causes of respiratory symptoms. Distinguishing spirometry-defined reversible airflow obstruction from nonspecific wheezing or other SCD-related pulmonary dysfunction is therefore important for both patient safety and appropriate allocation of care.

Evidence concerning discordance in children with SCD remains limited, particularly in tropical and ethnically diverse settings. French Guiana has a substantial pediatric SCD population and combines high infectious exposure, marked environmental humidity, variable access to specialist respiratory assessment, and geographic inequalities in healthcare delivery. These characteristics may influence both respiratory symptom burden and the probability that asthma is clinically recognized before objective testing. Nevertheless, few studies have directly compared routine clinical identification with systematic spirometric assessment in this population.

The present study aimed to quantify discordance between routine clinical suspicion of asthma and an objective, spirometry-based classification among children with SCD in French Guiana. The primary comparator was reversible airflow obstruction rather than a definitive asthma diagnosis. The operational comparator and sensitivity analyses reported here were developed post hoc during peer-review revision to address limitations in the original composite classification.

## 2. Materials and Methods

### 2.1. Study Design and Setting

We conducted an observational secondary analysis of prospectively recorded routine-care data from children with SCD assessed between January and July 2025. Respiratory assessment was offered during clinically stable routine follow-up. The analytical extract retained place of residence but did not contain a reliable recruitment-center variable; this study is therefore described geographically rather than by recruiting site.

This study assessed the recognition gap between clinical suspicion recorded before testing and an operational classification based on baseline spirometry and bronchodilator reversibility.

French Guiana is a French overseas territory in South America with a substantial pediatric SCD population and geographically variable access to pulmonary-function testing. In the analytical extract, 100 children resided in Saint-Laurent-du-Maroni and 40 in Kourou; these counts represent residence and must not be interpreted as center-specific recruitment counts.

### 2.2. Study Population

Children aged 5–17 years with confirmed SCD were eligible when clinically stable and able to attempt spirometry. A standardized respiratory assessment was offered as part of routine follow-up rather than being restricted to children already suspected of asthma. Because the source screening log and the denominator of all eligible children were not retained, the completeness of uptake cannot be established, and the pathway should not be interpreted as complete systematic spirometric screening.

The diagnosis of sickle cell disease was established as part of routine care using hemoglobin electrophoresis, high-performance liquid chromatography, molecular testing, or a combination of these methods.

Children were not assessed during an acute vaso-occlusive crisis, acute chest syndrome, respiratory infection, fever, or any other acute illness likely to interfere with respiratory testing. Participants with technically uninterpretable spirometry or incomplete respiratory assessment were retained for descriptive analyses when appropriate but were classified separately in analyses requiring a definitive respiratory diagnosis.

### 2.3. Data Collection

Routine-care data had been recorded prospectively using standardized clinical documentation derived from medical records, caregiver interviews, and respiratory assessments performed during usual care. These data were subsequently extracted and analyzed for the present study.

Variables available for analysis included age, sex, place of residence, SCD genotype, hydroxyurea treatment, previous acute chest syndrome, hospitalization history, clinical suspicion of asthma recorded before testing, ISAAC-derived symptoms, baseline spirometry, and bronchodilator reversibility. Classification-specific ICS and SABA exposure could not be reported because medication fields were not sufficiently complete in the analytical dataset and were therefore excluded from comparative analyses.

Information regarding previous asthma recognition had been documented before interpretation of the respiratory assessment, allowing comparison between routine clinical recognition and the subsequent study classification.

### 2.4. Routine Clinical Recognition of Asthma

Routine clinical recognition of asthma was defined as the presence of a physician-documented diagnosis of asthma in the child’s medical record before inclusion in this study.

Children without an explicitly documented asthma diagnosis were considered not previously recognized as having asthma. The analytical extract did not contain sufficiently complete, structured SABA or inhaled-corticosteroid exposure fields for valid category-specific reporting; medication use was therefore not used to define clinical recognition or to explain normal spirometry.

This approach was used to distinguish a formal clinical diagnosis from isolated respiratory symptoms or empirical treatment trials.

### 2.5. Respiratory Symptom Assessment

Respiratory symptoms were assessed using a standardized questionnaire administered to the child and caregiver. The questionnaire explored wheezing, nocturnal cough, exercise-related respiratory symptoms, dyspnea, chest tightness, previous episodes of breathing difficulty, use of inhaled bronchodilators, and previous emergency consultations for respiratory complaints.

Questions derived from the International Study of Asthma and Allergies in Childhood questionnaire were included to standardize the identification of asthma-compatible symptoms.

Symptom-based suspicion of asthma was analyzed separately from both previous physician-documented clinical recognition and the operational spirometry-based respiratory classification.

### 2.6. Spirometry

Spirometry was performed by trained healthcare personnel while the children were clinically stable, using standardized procedures consistent with international recommendations.

Participants were instructed and encouraged to perform repeated forced expiratory maneuvers until acceptable and reproducible measurements were obtained whenever possible. The principal spirometric variables recorded were forced expiratory volume in one second, forced vital capacity, and the forced expiratory volume in one second to forced vital capacity ratio.

Spirometry was performed with a MicroLab spirometer by trained personnel. The post hoc operational analysis used the variables retained in the routine-care extract: normal spirometry, FEV_1_/FVC < 80%, and bronchodilator reversibility. Height was not retained in the analytical dataset; consequently, GLI reference values or z-scores could not be reconstructed. Formal quality grades, rejected-maneuver counts, and numerical pre- and post-spirometric values were also unavailable.

### 2.7. Bronchodilator Reversibility Testing

Children with a recorded baseline spirometric result underwent bronchodilator testing as part of the screening pathway.

Salbutamol 400 µg was administered through a spacer device, and spirometry was repeated 15 min later. A positive bronchodilator response was defined as an increase in FEV_1_ of at least 12% from baseline. Bronchodilator testing supported the operational identification of variable expiratory airflow limitation.

### 2.8. Post Hoc Operational Definition of Reversible Airflow Obstruction

During peer-review revision, airflow obstruction was defined operationally as FEV_1_/FVC < 80%, and a positive bronchodilator response as an FEV_1_ increase ≥12% from baseline 15 min after salbutamol 400 µg delivered through a spacer. The post hoc objective category, termed spirometry-defined reversible airflow obstruction, required both obstruction and a positive bronchodilator response. Symptoms were analyzed descriptively and were not incorporated into this comparator.

This operational comparator identifies reversible obstruction but is not a diagnostic gold standard for asthma. A definitive asthma diagnosis requires integration of symptoms, repeated objective testing, treatment response, and longitudinal clinical assessment.

Participants were assigned post hoc to three mutually exclusive analytical categories: reversible obstruction; normal spirometry without obstruction or reversibility; and unclassified respiratory status. Re-audit of the participant-level data showed that this derivation changed membership relative to the legacy ‘asthma confirmed’ flag despite leaving the total case count at 43: REC-060 and REC-123 left the case group because obstruction was recorded without reversibility, whereas REC-019 and REC-026 entered because both obstruction and reversibility were recorded. The unclassified category comprised 70 children for whom none of the extract’s objective classification fields yielded a usable category and two with obstruction without documented reversibility. The extract did not preserve whether the 70 reflected technically unacceptable maneuvers, absent post-bronchodilator testing, or incomplete data capture, so these mechanisms could not be separated.

### 2.9. Post Hoc Respiratory Classification

The three-category post hoc respiratory classification was used only for the revised analyses. No child had isolated bronchodilator reversibility without baseline obstruction in the retained classification fields. Two children had baseline obstruction without documented reversibility and were retained as an intermediate unclassified pattern.

Reversible obstruction without prior clinical suspicion was defined as spirometry-defined reversible airflow obstruction in a child without clinical suspicion documented before standardized testing.

Within the reversible-obstruction group, participants were further described as previously suspected or not previously suspected. Additional details of the operational classification and supporting reporting materials are provided in the Supplementary Materials (Figure S1; Tables S1–S3; Checklists S1 and S2).

### 2.10. Study Outcomes

The primary outcome was discordance between clinical suspicion recorded before testing and spirometry-defined reversible airflow obstruction among participants with a definitive objective classification.

Secondary outcomes included the sample proportions in each respiratory category, the proportion of reversible-obstruction cases without prior clinical suspicion, agreement and classification measures, characteristics of classified versus unclassified participants, and exploratory characteristics of suspected versus previously unsuspected reversible obstruction.

### 2.11. Statistical Analysis

Continuous variables were summarized using means and standard deviations or medians and interquartile ranges, according to their distribution. Categorical variables were presented as frequencies and percentages.

Baseline characteristics were compared between classified and unclassified participants and, in an additional exploratory comparison requested during peer review, between children with reversible obstruction and those with normal spirometry. Within the reversible-obstruction group, children with and without prior clinical suspicion were also compared. Continuous variables were compared using Welch’s *t* test or the Mann–Whitney U test, and categorical variables were compared using Fisher’s exact test.

The post hoc primary concordance analysis excluded unclassified participants and deliberately compared the two unequivocal spirometric extremes: 43 children with reversible obstruction and 25 with normal spirometry. Sensitivity, specificity, predictive values, likelihood ratios, and Cohen’s kappa were calculated with 95% confidence intervals. Because this two-gate design excludes intermediate or incomplete patterns and can inflate apparent discrimination, specificity and predictive values are interpreted only within this restricted analytical subset and not as general screening-performance characteristics. Accuracy was omitted from the primary presentation because it is prevalence-dependent and difficult to interpret in this constructed subset.

Exact Clopper–Pearson 95% confidence intervals were used for proportions and classification measures. Likelihood-ratio confidence intervals used the log method, and the kappa interval was obtained by nonparametric bootstrap with 20,000 resamples and a fixed reproducibility seed.

All comparisons outside the primary concordance table were exploratory and unadjusted; no multivariable model was fitted, and no multiplicity adjustment was applied. *p* values are therefore interpreted descriptively rather than as confirmatory evidence.

All statistical tests were two-sided. Analyses were performed using Stata version 17 (StataCorp LLC, College Station, TX, USA).

### 2.12. Handling of Missing and Unclassifiable Data

The number of available observations was reported for each analysis. The primary concordance analysis included participants with both prior clinical-suspicion data and a definitive objective classification.

Participants without a usable post hoc objective classification or with an intermediate spirometric pattern were retained in the unclassified category and excluded from the primary two-gate comparison. Among 72 unclassified children, 70 had no usable objective category in the retained extract, and two had obstruction without documented reversibility. The source extract did not permit the 70 to be subdivided into technically unacceptable spirometry, missing post-bronchodilator testing, or data-extraction gaps.

No imputation was performed. The primary comparison was a complete-case two-gate analysis. The extreme-case analyses that alternatively assigned all 72 unclassified participants to the outcome-negative or outcome-positive category were post hoc sensitivity analyses developed during revision and are presented only to illustrate sensitivity to missing classification.

### 2.13. Ethical Considerations

This study was conducted in accordance with the Declaration of Helsinki and the French framework applicable to secondary use of routine-care health data. The work was conducted within the broader DREPASTHME framework (version 1.0, 22 March 2024), for which the Centre Hospitalier de Cayenne served as data controller and data processing followed the CNIL MR-004 reference methodology.

No research-specific examination, intervention, biological sampling, or additional visit was performed for this secondary analysis. The dataset was pseudonymized, and access was restricted to authorized personnel. Under Article L.1121-1 of the French Public Health Code, this secondary analysis did not constitute research involving the human person requiring review by a Comité de Protection des Personnes. Parents or legal guardians were informed, through the institutional information procedure, of possible secondary use of routine-care data and their right to object. A study-specific MR-004 declaration number or Health Data Hub identifier was not available in the analytical materials and is therefore not reported.

## 3. Results

### 3.1. Study Population and Respiratory Classification

Routine-care data from 140 children with SCD were analyzed. Mean age was 10.3 ± 3.8 years; 76 (54.3%) were female, 105 (75.0%) had HbSS, 104 (74.3%) lived in rural settings, and 107 (76.4%) received hydroxyurea. Residence was Saint-Laurent-du-Maroni for 100 children and Kourou for 40; the extract did not retain a reliable recruitment-center variable.

Under the post hoc operational derivation, reversible airflow obstruction was identified in 43 children (30.7%), 25 (17.9%) had normal spirometry without obstruction or reversibility, and 72 (51.4%) were unclassified. Of the 72, 70 had no usable objective classification in the retained extract, and 2had obstruction without documented reversibility; no isolated reversibility-without-obstruction pattern was identified. The source data did not distinguish technical test failure from missing post-bronchodilator testing orincomplete data capture among the 70. Baseline characteristics of classified and unclassified participants are presented in Table 1.

**Figure 1:**
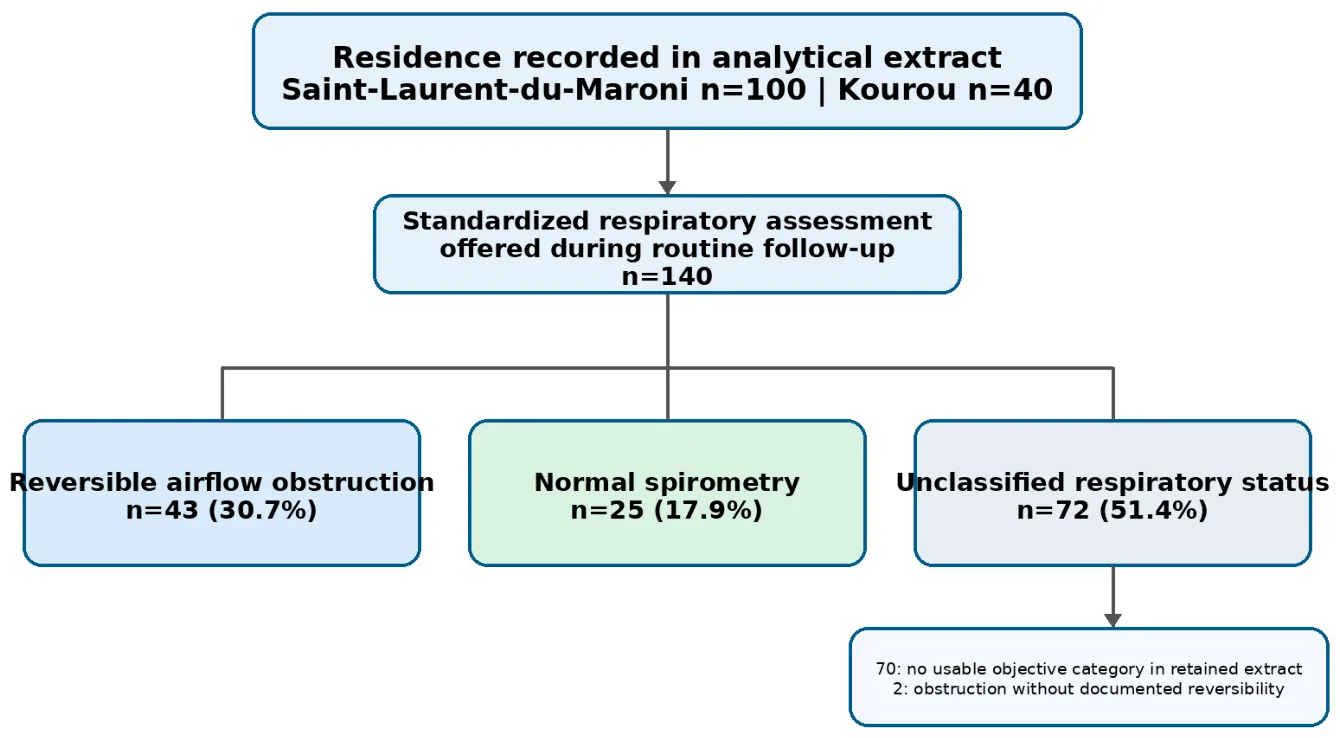
Participant flow and post hoc mutually exclusive respiratory classification among 140 children with SCD. Residence counts reflect the analytical residence variable and are not recruitment-center counts. Reversible obstruction required FEV_1_/FVC < 80% and FEV_1_ improvement ≥12% after bronchodilator. Unclassified status comprised 70 records without a usable objective category in the retained extract and two cases of obstruction without documented reversibility.

The mutually exclusive respiratory classification and reasons for non-classification are shown in Figure 1.

### 3.2. Discordance Between Routine Clinical Recognition and Standardized Respiratory Assessment

Clinical suspicion of asthma had been recorded before testing in 35 children (25.0%): 10/43 (23.3%) with reversible obstruction, 3/25 (12.0%) with normal spirometry, and 22/72 (30.6%) who remained unclassified. Conversely, 33/43 (76.7%) children with reversible obstruction had no prior suspicion.

Prior clinical suspicion was present in fewer than one-quarter of children with reversible obstruction. Table 2 presents the complete 3 × 2 cross-tabulation.

In the deliberately contrastive classified-only analysis (*n* = 68), clinical suspicion had sensitivity 23.3% (95% CI 11.8–38.6), specificity 88.0% (68.8–97.5), PPV 76.9% (46.2–95.0), NPV 40.0% (27.0–54.1), LR+ 1.94 (0.59–6.39), and LR− 0.87 (0.70–1.09). Agreement was slight (κ = 0.090; 95% CI −0.059 to 0.239). These estimates describe only the restricted comparison of unequivocally positive and negative spirometric patterns.

The likelihood-ratio confidence intervals were wide and included 1.0. The point estimates were directionally compatible with some information in prior clinical suspicion, but the small number of suspicion-positive classified participants (*n* = 13) produced substantial imprecision, and the data cannot establish either useful discrimination or absence of discrimination.

**Figure 2:**
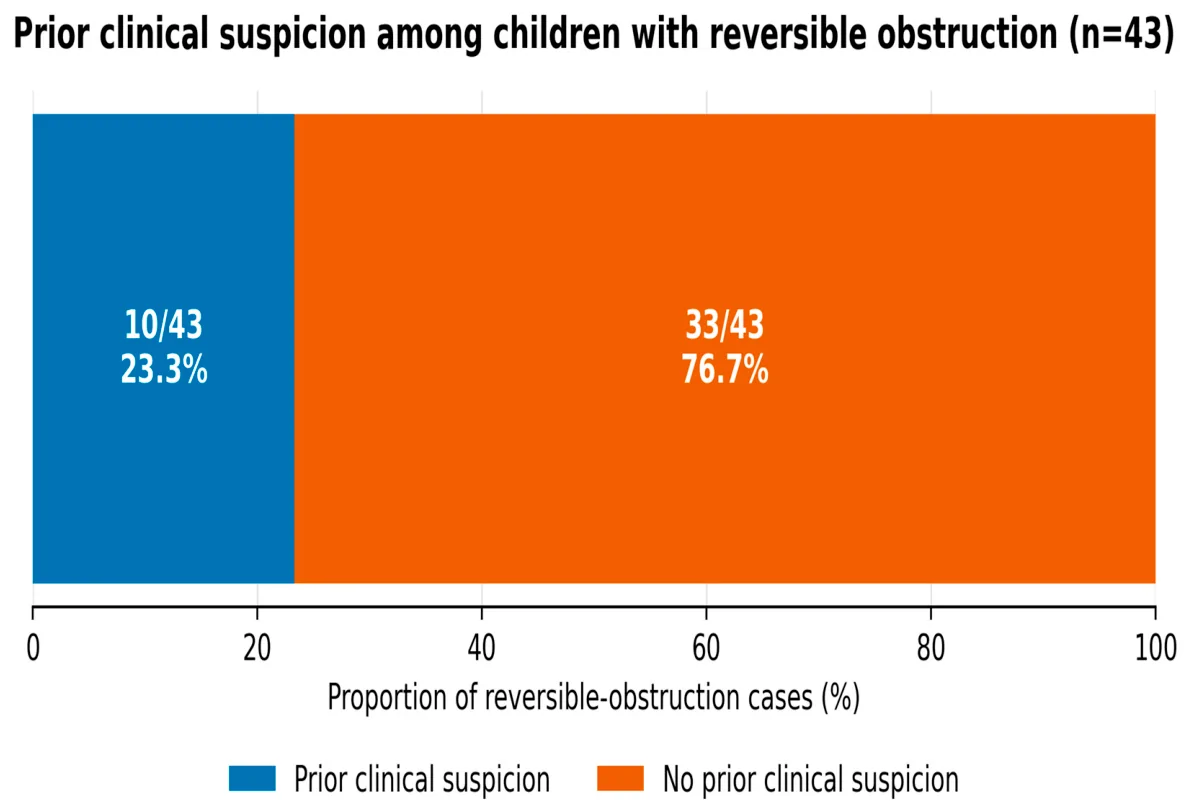
Previous clinical suspicion among children with spirometry-defined reversible obstruction. Ten (23.3%) had prior suspicion and 33 (76.7%) did not.

Figure 2 shows prior clinical suspicion among the 43 children with reversible obstruction.

### 3.3. Primary and Sensitivity Analyses

ISAAC-derived categories were not evaluated as diagnostic tests because symptom questions were designed for screening and the objective comparator was a single spirometric assessment. ISAAC 2–3 was positive in 30/140 (21.4%) and ISAAC 4–5 in 110/140 (78.6%); among the 43 children with reversible obstruction, the corresponding counts were 8 (18.6%) and 33 (76.7%).

Table 3 reports the post hoc complete-case comparison and the two extreme sensitivity analyses developed during revision.

Results varied materially according to the handling of unclassified participants. Because unclassified children had more previous acute chest syndrome than classified children (43.1% vs. 25.0%, *p* = 0.032), missing objective classification was not randomly distributed with respect to prior pulmonary morbidity.

**Figure 3:**
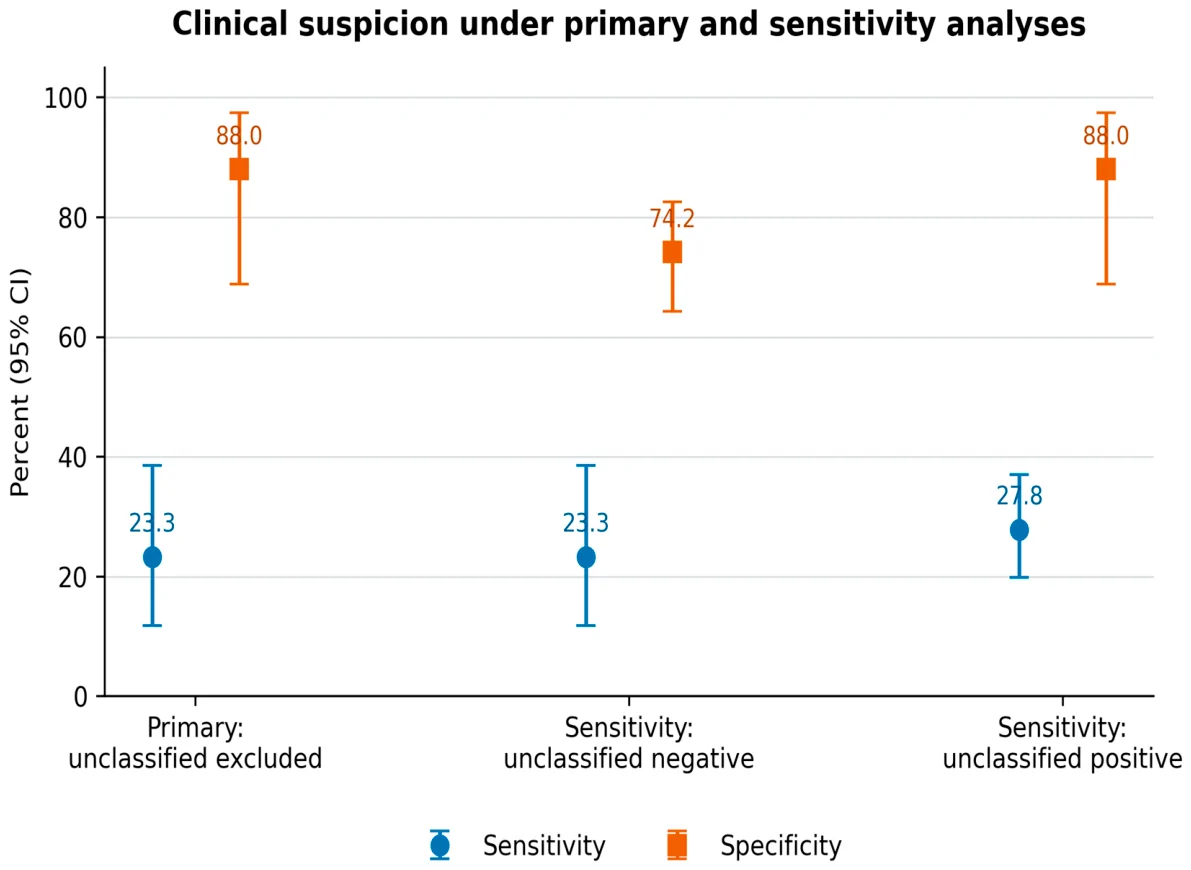
Sensitivity and specificity of prior clinical suspicion with 95% confidence intervals under the primary analysis and two extreme assumptions for unclassified participants.

Figure 3 compares sensitivity and specificity across the primary and sensitivity analyses.

### 3.4. Characteristics of Reversible Obstruction According to Prior Clinical Suspicion

Among the 43 children with reversible obstruction, 10 had prior clinical suspicion, and 33 had no previous suspicion. Their characteristics are presented in Table 4.

Children without prior suspicion had more previous hospitalizations than those with prior suspicion (median 3 [IQR 2–4] versus 2 [2]; *p* = 0.002). All children with prior suspicion received hydroxyurea versus 21/33 (63.6%) without prior suspicion (*p* = 0.040). These exploratory comparisons were unadjusted and were not corrected for multiplicity. The analytical extract retained hospitalization counts but not the indication for each admission; therefore, cause-specific hospitalization analyses could not be performed.

No statistically significant differences were identified for age, sex, genotype, residence, previous acute chest syndrome, tobacco-smoke exposure, rhinitis, eczema, or ISAAC responses. Classification-specific ICS and SABA data were not sufficiently complete for valid reporting and were excluded rather than imputed.

### 3.5. Main Findings

The reanalysis identified substantial discordance: 76.7% of children with reversible obstruction had no prior clinical suspicion. However, 51.4% of the cohort remained unclassified, and performance estimates changed with assumptions about this group.

ISAAC categories were retained as descriptive screening measures rather than diagnostic-performance indices. The primary comparison showed low sensitivity, high but imprecise specificity, and only slight agreement.

## 4. Discussion

The principal finding was substantial discordance between prior clinical suspicion and spirometry-defined reversible obstruction in children with SCD. Only 10/43 children with reversible obstruction had been suspected before testing, while 33 (76.7%) had not. In the restricted two-gate analysis, sensitivity was low, and agreement was slight (κ = 0.090). LR+ and LR− point estimates were in the expected direction, but their confidence intervals were wide and included unity, so this study demonstrates imprecision rather than absence of discrimination.

The high proportion of previously unsuspected reversible obstruction is clinically relevant, but reversible obstruction is not synonymous with a definitive asthma diagnosis. Current pediatric asthma guidance emphasizes compatible history, objective evidence, and longitudinal clinical confirmation [6,8,11,12].

The diagnostic challenge is likely greater in SCD because wheezing, cough, dyspnea, exercise limitation, chest discomfort, and nocturnal symptoms are not specific to asthma. These manifestations may result from anemia, recurrent respiratory infections, previous acute chest syndrome, airway inflammation, pulmonary vascular dysfunction, deconditioning, or progressive SCD-related lung disease. Recent work has shown that children with SCD and asthma may have increased airway resistance or inflammatory abnormalities even when conventional spirometric indices do not clearly distinguish them from children with SCD without asthma [16,17]. Thus, clinical symptoms and spirometric findings may reflect overlapping but not identical respiratory mechanisms.

The 30.7% sample proportion of reversible obstruction exceeded prior clinical recognition, but it must not be interpreted as population prevalence because the denominator of all followed children was unavailable, and the analysis was restricted to screened participants. Pulmonary-function studies in SCD have reported obstructive, restrictive, mixed, and unclassified patterns [18,19,20,21,22].

The low sensitivity of prior clinical suspicion indicates that absence of an asthma label cannot exclude reversible obstruction. However, the primary comparison deliberately contrasted unequivocal reversible obstruction with unequivocally normal spirometry and removed intermediate or incomplete patterns. This two-gate design tends to favor discrimination, particularly specificity. That agreement nevertheless remained slight under these favorable conditions strengthens the conclusion that prior clinical suspicion and the spirometric phenotype were poorly aligned, while the wide confidence intervals prevent precise estimation of diagnostic performance.

Objective testing must also be interpreted cautiously. A normal spirometry result during a clinically stable period does not exclude intermittent asthma, while isolated obstruction or bronchodilator responsiveness does not automatically establish the diagnosis. Pulmonary function should be interpreted using age-appropriate reference limits and clinical context [11,23]. A GLI-based sensitivity analysis could not be performed because height was not retained in the analytical extract. The updated ATS/ERS approach to bronchodilator responsiveness may also classify some children differently from older percentage-change thresholds [24].

ISAAC-derived responses were analyzed descriptively rather than as diagnostic tests. The broader ISAAC 4–5 category was positive in most children across all respiratory categories, illustrating the high background burden and limited specificity of symptom screening in SCD.

In SCD, symptom-questionnaire performance may be particularly affected by the high background prevalence of respiratory complaints. Asthma-screening studies in children with SCD show that questionnaire findings require objective follow-up rather thanimmediate diagnostic labeling [13,14]. Questionnaire-based assessment has also been evaluated for respiratory tract infections in children with SCD [25].

Three children with prior clinical suspicion had normal spirometry, and twenty-two remained unclassified. These findings do not prove that the original suspicion was incorrect. Because inhaled-treatment exposure was not sufficiently complete for category-specific analysis, treatment-induced normalization cannot be evaluated as an explanation in these children. Longitudinal reassessment is therefore essential.

This distinction is especially important in SCD because both undertreatment and overtreatment may have consequences. Missing clinically relevant asthma could increase exposure to recurrent symptoms, hypoxemia, emergency visits, and pulmonary complications. Conversely, attributing all respiratory manifestations to asthma could result in unnecessary medication and delayed investigation of other conditions such as sleep-disordered breathing, chronic hypoxemia, restrictive lung disease, infection, or evolving SCD-related pulmonary damage.

Asthma identification has potential prognostic relevance because pulmonary comorbidity may contribute to acute chest syndrome and other serious outcomes. A meta-analysis supports an association between asthma and acute chest syndrome in SCD [4], while practical and systematic reviews emphasize the clinical overlap and need for careful management [26,27]. Corticosteroid use may improve selected respiratory outcomes but has also been associated with rebound pain or readmission, underscoring the importance of accurate respiratory classification [28].

The higher hospitalization count among children with reversible obstruction but no prior suspicion is potentially important, although it was an exploratory, unadjusted finding and should not be interpreted causally. The analytical extract did not retain admission-level indications, so hospitalizations could not be categorized as vaso-occlusive pain, acute chest syndrome, hypoxia, infection, or asthma-related events. Recurrent hospital contact should nevertheless provide an opportunity to reassess respiratory symptoms and request objective testing [29].

The absence of clear differences in rhinitis, eczema, tobacco exposure, genotype, or previous acute chest syndrome between children with reversible obstruction with and without prior clinical suspicion further suggests that the absence of prior recognition cannot be predicted reliably from a small number of conventional clinical characteristics. Symptom-control questionnaires are useful after asthma has been diagnosed, but their validity and interpretation vary across ages, languages, cultures, and populations [30]. Respiratory screening in French Guiana may additionally be influenced by linguistic diversity, environmental exposures, geographic barriers, and differences in healthcare access.

The clinical implications of our findings are therefore practical. Children with SCD should undergo systematic respiratory questioning during routine follow-up, but positive symptoms should trigger rather than replace standardized study assessment. Spirometry with bronchodilator reversibility should be considered in school-aged children with recurrent wheezing, exercise limitation, nocturnal symptoms, unexplained cough, repeated respiratory emergencies, or previous acute chest syndrome. Functional limitation and reduced quality of life may otherwise remain insufficiently recognized in children with SCD [31].

Integration of hematology and pulmonary care could improve respiratory assessment. An integrated pediatric SCD–pulmonary clinic has been shown to be feasible [32], and pulmonary subspecialty care has been associated with reduced subsequent acute respiratory healthcare use [15]. Recent data demonstrate variability in asthma-controller use among children with SCD [33], while contemporary reviews support proactive assessment of pulmonary comorbidity as part of comprehensive SCD care [34].

Strengths include prospectively recorded routine-care data, direct comparison with pre-test clinical suspicion, explicit separation of unclassified participants, participant-level re-audit of the revised operational derivation, and inclusion of an underrepresented tropical pediatric SCD population.

Several limitations should be acknowledged. First, the operational comparator and sensitivity analyses were developed post hoc during peer-review revision, and reversible obstruction at one visit is not a diagnostic gold standard for asthma. Second, the primary analysis used a two-gate design comparing 43 unequivocal reversible-obstruction cases with 25 unequivocally normal spirometries; exclusion of intermediate or incomplete patterns can inflate discrimination, particularly specificity. Third, 72/140 participants (51.4%) remained unclassified. Unclassified children had more previous acute chest syndrome than classified children (43.1% vs. 25.0%, *p* = 0.032), indicating that missing classification was concentrated in children with greater prior pulmonary morbidity and may bias the complete-case analysis. The extract could not distinguish technical spirometry failure from absent post-bronchodilator testing or incomplete data capture among 70 of these children. Fourth, the denominator and characteristics of all eligible but unassessed children were unavailable, so 30.7% is a sample proportion rather than a prevalence estimate. Fifth, a fixed FEV_1_/FVC < 80% threshold was used; height was absent, preventing reconstruction of GLI z-scores or lower limits of normal. Sixth, FeNO, eosinophils, IgE, skin-prick testing, bronchial provocation, peak-flow variability, and oscillometry were unavailable. Seventh, admission indications and sufficiently complete inhaled-treatment data were unavailable. Eighth, the extract contained residence but not a reliable recruitment-center variable. Finally, re-audit demonstrated that the legacy case flag and the revised obstruction-plus-reversibility algorithm selected different participants even though both yielded *n* = 43; this derivation change is now reported explicitly.

## 5. Conclusions

Prior clinical suspicion showed low sensitivity and slight agreement with spirometry-defined reversible airflow obstruction in the restricted classified subset. The likelihood-ratio estimates were imprecise, and the primary two-gate comparison should not be interpreted as a general diagnostic-performance study. More than half of participants lacked a usable objective classification, with greater previous acute chest syndrome among unclassified children, creating potential selection bias. These findings support improved access to complete, quality-assured, longitudinal respiratory assessment rather than reliance on clinical suspicion or a single spirometric result.

## Figures and Tables

**Table 1:** Baseline characteristics of classified and unclassified participants.

**(A)**												
**Characteristic**	**Overall, *n* = 140**			**Classified, *n* = 68**			**Unclassified, *n* = 72**			***p* Value**		
Age, years, mean ± SD	10.3 ± 3.8			10.4 ± 3.8			10.2 ± 3.8			0.669		
Female sex	76 (54.3)			35 (51.5)			41 (56.9)			0.611		
HbSS genotype	105 (75.0)			51 (75.0)			54 (75.0)			1.000		
Rural residence	104 (74.3)			56 (82.4)			48 (66.7)			0.052		
Residence in Saint-Laurent-du-Maroni	100 (71.4)			46 (67.6)			54 (75.0)			0.356		
Hydroxyurea treatment	107 (76.4)			50 (73.5)			57 (79.2)			0.551		
Previous acute chest syndrome	48 (34.3)			17 (25.0)			31 (43.1)			0.032		
Exposure to tobacco smoke	22 (15.7)			11 (16.2)			11 (15.3)			1.000		
Rhinitis symptoms	112 (80.0)			53 (77.9)			59 (81.9)			0.673		
Eczema symptoms	19 (13.6)			12 (17.6)			7 (9.7)			0.219		
Clinical suspicion in medical file	35 (25.0)			13 (19.1)			22 (30.6)			0.171		
ISAAC 2–3 positive	30 (21.4)			17 (25.0)			13 (18.1)			0.410		
ISAAC 4–5 positive	110 (78.6)			53 (77.9)			57 (79.2)			1.000		
Previous hospitalizations, median [IQR]	3 [2–4]			3 [2–4]			3 [2–4]			0.090		
**(B)**												
**Characteristic**	**Reversible Obstruction, *n* = 43**				**Normal Spirometry, *n* = 25**				***p* Value**			
Age, years, mean ± SD	10.6 ± 3.9				10.2 ± 3.7				0.641			
Female sex	22 (51.2)				13 (52.0)				1.000			
HbSS genotype	33 (76.7)				18 (72.0)				0.773			
Rural residence	38 (88.4)				18 (72.0)				0.108			
Residence in Saint-Laurent-du-Maroni	28 (65.1)				18 (72.0)				0.602			
Hydroxyurea treatment	31 (72.1)				19 (76.0)				0.783			
Previous acute chest syndrome	10 (23.3)				7 (28.0)				0.773			
Exposure to tobacco smoke	7 (16.3)				4 (16.0)				1.000			
Rhinitis symptoms	32 (74.4)				21 (84.0)				0.545			
Eczema symptoms	10 (23.3)				2 (8.0)				0.187			
Clinical suspicion in medical file	10 (23.3)				3 (12.0)				0.345			
ISAAC 2–3 positive	8 (18.6)				9 (36.0)				0.148			
ISAAC 4–5 positive	33 (76.7)				20 (80.0)				1.000			
Previous hospitalizations, median [IQR]	3 [2–4]				3 [2,3]				0.388			

Data are *n* (%) unless otherwise indicated. Classified participants comprised 43 with reversible obstruction and 25 with normal spirometry. *p* values compare classified with unclassified participants using Welch’s *t* test, Mann–Whitney U test, or Fisher’s exact test; analyses were exploratory and not adjusted for multiplicity. An additional exploratory comparison of reversible obstruction versus normal spirometry is provided in Table 1B; associations from the previous composite-asthma framing should not be carried forward to the revised respiratory classification.

**Table 2:** Prior clinical suspicion by mutually exclusive respiratory classification.

Prior Clinical Suspicion	Reversible Obstruction	Normal Spirometry	Unclassified	Total
Yes	10	3	22	35
No	33	22	50	105
Total	43	25	72	140

**Table 3:** Performance of prior clinical suspicion in the primary and sensitivity analyses.

Analysis	Sensitivity, % (95% CI)	Specificity, % (95% CI)	PPV, % (95% CI)	NPV, % (95% CI)	Cohen κ (95% CI)
Primary: unclassified excluded	23.3 (11.8–38.6)	88.0 (68.8–97.5)	76.9 (46.2–95.0)	40.0 (27.0–54.1)	0.090 (−0.059–0.239)
Sensitivity: unclassified negative	23.3 (11.8–38.6)	74.2 (64.3–82.6)	28.6 (14.6–46.3)	68.6 (58.8–77.3)	−0.027 (−0.183–0.139)
Sensitivity: unclassified positive	27.8 (19.9–37.0)	88.0 (68.8–97.5)	91.4 (76.9–98.2)	21.0 (13.6–30.0)	0.070 (−0.003–0.145)

PPV, positive predictive value; NPV, negative predictive value; CI, confidence interval. The primary analysis compared reversible obstruction with normal spirometry. Sensitivity analyses treated all unclassified participants as outcome-negative or outcome-positive.

**Table 4:** Characteristics of children with reversible obstruction according to previous clinical suspicion.

Characteristic	Prior Suspicion, *n* = 10	No Prior Suspicion, *n* = 33	*p* Value
Age, years, mean ± SD	11.1 ± 3.3	10.5 ± 4.0	0.615
Female sex	7 (70.0)	15 (45.5)	0.281
HbSS genotype	10 (100.0)	23 (69.7)	0.084
Rural residence	10 (100.0)	28 (84.8)	0.320
Residence in Saint-Laurent-du-Maroni	5 (50.0)	23 (69.7)	0.281
Hydroxyurea treatment	10 (100.0)	21 (63.6)	0.040
Previous acute chest syndrome	1 (10.0)	9 (27.3)	0.407
Tobacco-smoke exposure	2 (20.0)	5 (15.2)	0.656
Rhinitis symptoms	8 (80.0)	24 (72.7)	1.000
Eczema symptoms	1 (10.0)	9 (27.3)	0.407
ISAAC 2–3 positive	2 (20.0)	6 (18.2)	1.000
ISAAC 4–5 positive	7 (70.0)	26 (78.8)	0.674
Previous hospitalizations, median [IQR]	2 [2]	3 [2–4]	0.002

## Data Availability

The datasets generated and analyzed during the current study are not publicly available because they contain potentially sensitive clinical information relating to children with sickle cell disease. De-identified data may be made available by the corresponding author upon reasonable request and subject to approval by the responsible institution and compliance with applicable French data-protection regulations.

## Supplementary Materials

The following supporting information can be downloaded at: https://www.mdpi.com/article/10.3390/jcm15166342/s1, Figure S1: Operational respiratory-classification algorithm; Table S1: Reasons for unclassified respiratory status; Table S2: Clinical-suspicion cross-tabulation across the three categories; Table S3: Primary and sensitivity analyses; Checklist S1: STROBE checklist; Checklist S2: STARD 2015 checklist.

## Abbreviations

ACS	acute chest syndrome
ATS	American Thoracic Society
CI	confidence interval
ERS	European Respiratory Society
FEV_1_	forced expiratory volume in one second
FVC	forced vital capacity
HbF	fetal hemoglobin
ISAAC	International Study of Asthma and Allergies in Childhood
NPV	negative predictive value
OR	odds ratio
PPV	positive predictive value
SCD	sickle cell disease
SD	standard deviation
VOC	vaso-occlusive crisis
