# Supplementary material for: Discordance Between Clinical Suspicion and Spirometry-Defined Reversible Airflow Obstruction in Children with Sickle Cell Disease in French Guiana
Source: J Clin Med. 2026 Aug 17;15(16):6342. doi: 10.3390/jcm15166342 (PMC13513272; doi:10.3390/jcm15166342)
Supplement: Supplementary file 1 [file jcm-15-06342-s001.zip › JCM_4488223_Checklist_S1_STROBE.pdf]

Journal of Clinical Medicine (JCM) – Supplementary Material

Discordance Between Clinical Suspicion and Spirometry-Defined Reversible Airflow Obstruction in Children with Sickle Cell Disease in French Guiana

Manuscript ID: jcm-4488223

Gabriel Bafunyembaka, Estiverne Evens, Nadia Nathan, Arriel Makembi, Narcisse Elenga

Checklist S1. STROBE checklist for cross-sectional observational reporting

This checklist maps the final JCM proof to the STROBE cross-sectional reporting domains. Requirement wording is paraphrased for concise manuscript-specific completion.

Item	Section/topic	Reporting requirement (paraphrased)	Location in manuscript	Completion/comment
1a	Title and abstract	Indicate the observational study design in the title or abstract.	Abstract, Methods	Yes – the abstract describes prospectively recorded routine-care data and the post hoc secondary analysis; the title emphasizes the clinical/spirometric comparison.
1b	Title and abstract	Provide a balanced summary of methods and findings.	Abstract	Yes – population, comparator, group sizes, accuracy measures with CIs, and key limitations are summarized.
2	Background/rationale	Explain the scientific background and rationale.	Introduction, pp. 2–4	Yes – respiratory morbidity, diagnostic overlap in SCD, and need for objective assessment are described.
3	Objectives	State specific objectives and any prespecified hypotheses.	End of Introduction, p. 4	Yes – objective is to quantify discordance between routine clinical suspicion and spirometry-based classification; post hoc nature of comparator is disclosed.
4	Study design	Present key elements of study design early.	Methods 2.1, p. 5	Yes – observational secondary analysis of prospectively recorded routine-care data.
5	Setting	Describe setting, locations, relevant dates, and periods of data collection.	Methods 2.1, p. 5	Yes – French Guiana; January–July 2025; residence counts for Saint-Laurent-du-Maroni and Kourou; recruitment-center variable limitation stated.
6a	Participants	Give eligibility criteria and methods of participant selection.	Methods 2.2, p. 5	Yes – age 5–17 years, confirmed SCD, clinically stable, able to attempt spirometry; respiratory assessment offered during routine follow-up.
6b	Participants	For matched studies, report matching criteria and numbers.	Not applicable	Not applicable – no matching was used.
7	Variables	Clearly define outcomes, exposures, predictors, confounders, and diagnostic criteria.	Methods 2.3–2.10, pp. 5–7	Yes – prior clinical recognition, symptom measures, spirometry, reversibility, three-category classification, and outcomes are defined.
8	Data sources/measurement	For each variable, give sources and measurement methods.	Methods 2.3–2.7, pp. 5–6	Yes – medical records/caregiver interviews, questionnaire, MicroLab spirometry, salbutamol 400 µg via spacer, repeat at 15 min.
9	Bias	Describe efforts to address potential sources of bias.	Methods 2.8, 2.11–2.12; Discussion limitations	Yes – post hoc derivation, two-gate restriction, unclassified group, sensitivity analyses, and selection/missing-classification biases are explicitly addressed.
10	Study size	Explain how study size was arrived at.	Methods 2.2; Results 3.1	The analysis included all 140 available

Item	Section/topic	Reporting requirement (paraphrased)	Location in manuscript	Completion/comment
				assessed children in the retained extract. No prespecified sample-size calculation is reported; denominator of all eligible children was unavailable.
11	Quantitative variables	Explain handling of quantitative variables and category boundaries.	Methods 2.6–2.8, 2.11	Yes – FEV_1/FVC <80%, bronchodilator FEV_1 increase ≥12%, and continuous/categorical summary methods are stated.
12a	Statistical methods	Describe all statistical methods.	Methods 2.11, pp. 7–8	Yes – Welch t test, Mann–Whitney U, Fisher exact test, classification measures, CI methods, bootstrap kappa, Stata 17.
12b	Statistical methods	Describe subgroup/interaction methods.	Methods 2.11; Results 3.4	Exploratory classified vs unclassified, reversible obstruction vs normal, and suspected vs unsuspected comparisons are described; no interaction modeling was performed.
12c	Statistical methods	Explain how missing data were addressed.	Methods 2.12, p. 8	Yes – complete-case two-gate primary analysis, no imputation, 72 unclassified retained separately, extreme-case sensitivity analyses.
12d	Statistical methods	If relevant, describe methods accounting for sampling strategy.	Methods 2.1–2.2; limitations	No probability sampling or center-cluster analysis was possible; recruitment-center variable and complete eligible denominator were unavailable.
12e	Statistical methods	Describe sensitivity analyses.	Methods 2.12; Results 3.3	Yes – all unclassified treated as outcome-negative or outcome-positive.
13a	Participants	Report numbers at each stage of the study.	Results 3.1; Figure 1	Yes – n=140; 43 reversible obstruction, 25 normal spirometry, 72 unclassified.
13b	Participants	Give reasons for non-participation/non-classification at each stage.	Methods 2.2, 2.12; Results 3.1; Table S1	Reasons for unclassified status are reported to the extent supported by the extract; denominator/reasons for eligible but unassessed children were not retained.
13c	Participants	Consider a flow diagram.	Figure 1; Supplementary Figure S1	Yes.
14a	Descriptive data	Give participant characteristics and relevant exposures/confounders.	Table 1; Results 3.1	Yes – age, sex, genotype, residence, hydroxyurea, ACS, tobacco, rhinitis, eczema, clinical suspicion, ISAAC, hospitalization history.
14b	Descriptive data	Indicate numbers with missing data for variables of interest.	Methods 2.12; Results 3.1; Table S1	Yes for objective classification: 72 unclassified, including 70 without a usable objective category and 2 with obstruction without documented reversibility; further mechanisms could not be separated.
14c	Descriptive data	For cohort studies, summarize follow-up time.	Not applicable	Not applicable – this is not a longitudinal cohort analysis.
15	Outcome data	Report numbers of outcome events or summary measures.	Results 3.1–3.3; Tables 2–3	Yes – respiratory categories, prior suspicion cross-tabulation, classification measures and CIs are reported.
16a	Main results	Give unadjusted/adjusted estimates and precision; identify confounders where relevant.	Results 3.2–3.4; Tables 3–4	Classification estimates have 95% CIs. Exploratory comparisons are explicitly unadjusted; no multivariable model was fitted.
16b	Main results	Report category boundaries when continuous variables are categorized.	Methods 2.7–2.8	Yes – FEV_1/FVC <80% and FEV_1 increase ≥12%.
16c	Main results	If relevant, translate relative risks to absolute risks.	Not applicable	Not applicable – no relative-risk model was used.
17	Other analyses	Report subgroup, interaction, and sensitivity analyses.	Results 3.3–3.4; Tables 3–4	Yes – extreme-case sensitivity analyses and exploratory subgroup comparisons are reported.
18	Key results	Summarize key results with reference to objectives.	Results 3.5; opening Discussion	Yes – 76.7% of reversible-obstruction cases lacked prior suspicion; performance estimates are qualified by the unclassified group.

Item	Section/topic	Reporting requirement (paraphrased)	Location in manuscript	Completion/comment
19	Limitations	Discuss limitations and potential bias/imprecision.	Discussion, pp. 16–17	Yes – post hoc comparator, two-gate design, 51.4% unclassified, missing denominator, fixed threshold, unavailable tests/treatments/admission indications, residence vs recruitment center.
20	Interpretation	Give a cautious overall interpretation.	Discussion; Conclusions	Yes – reversible obstruction is explicitly not equated with definitive asthma and results are framed as discordance rather than diagnostic proof.
21	Generalisability	Discuss external validity.	Discussion	Yes – tropical French Guiana context, care access/geography, and restricted screened sample are discussed.
22	Funding	Give funding source and role of funders.	Funding statement, p. 17	Yes – no specific grant from public, commercial, or not-for-profit sectors.

Note: STROBE is the primary reporting framework because this manuscript is an observational secondary analysis of routine-care data. Locations refer to the 20-page JCM proof provided for final proofreading; pagination may shift slightly after production.
