# Supplementary material for: Discordance Between Clinical Suspicion and Spirometry-Defined Reversible Airflow Obstruction in Children with Sickle Cell Disease in French Guiana
Source: J Clin Med. 2026 Aug 17;15(16):6342. doi: 10.3390/jcm15166342 (PMC13513272; doi:10.3390/jcm15166342)
Supplement: Supplementary file 1 [file jcm-15-06342-s001.zip › JCM_4488223_Checklist_S2_STARD_2015.pdf]

Journal of Clinical Medicine (JCM) – Supplementary Material

Discordance Between Clinical Suspicion and Spirometry-Defined Reversible Airflow Obstruction in Children with Sickle Cell Disease in French Guiana

Manuscript ID: jcm-4488223

Gabriel Bafunyembaka, Estiverne Evens, Nadia Nathan, Arriel Makembi, Narcisse Elenga

Checklist S2. STARD 2015 checklist (applicability-adapted)

Important applicability statement: the manuscript reports measures commonly used in diagnostic-accuracy studies, but the authors explicitly state that spirometry-defined reversible airflow obstruction is an operational comparator and not an independent reference standard for asthma. STARD items are therefore mapped transparently, with non-applicable or only partially applicable items identified rather than forced into a diagnostic-gold-standard framework.

Item	Section/topic	STARD 2015 requirement (paraphrased)	Location in manuscript	Completion/applicability comment
1	Title/abstract	Identify a diagnostic-accuracy study using at least one accuracy measure.	Title; Abstract	Partially applicable – the study is deliberately framed as discordance/concordance, not a general diagnostic-accuracy study. The abstract reports sensitivity, specificity, likelihood ratios, and κ with CIs.
2	Abstract	Provide a structured summary of design, methods, results, and conclusions.	Abstract	Yes.
3	Introduction	Describe scientific/clinical background, intended use, and clinical role of the index test.	Introduction, pp. 2–4	Yes – prior routine clinical suspicion is compared with objective spirometric assessment in pediatric SCD.
4	Introduction	State study objectives and hypotheses.	End of Introduction, p. 4	Yes – aim is to quantify discordance; operational comparator and sensitivity analyses are disclosed as post hoc.
5	Study design	State whether data collection was planned before index/comparator testing.	Methods 2.1 and 2.3	Data were recorded prospectively during routine care; the present secondary analysis and operational comparator were developed post hoc.
6	Participants	State eligibility criteria.	Methods 2.2	Yes – age 5–17, confirmed SCD, clinically stable, able to attempt spirometry.
7	Participants	Explain how potentially eligible participants were identified.	Methods 2.1–2.2	Respiratory assessment was offered during clinically stable routine follow-up; complete screening log/eligible denominator was not retained.
8	Participants	State setting, location, and dates of identification.	Methods 2.1	Yes – French Guiana; January–July 2025; residence distribution reported.
9	Participants	State whether the participant series was consecutive, random, or convenience-based.	Methods 2.2; limitations	The retained data do not establish complete uptake or a consecutive series; this limitation is explicitly reported.
10a	Index test	Describe the index test in enough detail to reproduce it.	Methods 2.4	Yes – prior physician-documented asthma diagnosis/clinical recognition in the medical record before study testing.
10b	Comparator	Describe the reference/comparator in enough detail to reproduce it.	Methods 2.6–2.9	Yes for the operational comparator: MicroLab spirometry; obstruction FEV_1/FVC <80%; salbutamol 400 µg via spacer; repeat at 15 min; FEV_1 increase ≥12%.
11	Comparator	Explain rationale for choosing the	Methods 2.8; Discussion	No independent diagnostic reference

Item	Section/topic	STARD 2015 requirement (paraphrased)	Location in manuscript	Completion/applicability comment
		reference standard when alternatives exist.		standard was used. The manuscript explicitly states that reversible obstruction is an operational comparator, not a gold standard for asthma.
12a	Index test	Define and justify positivity/categorical cutoffs for the index test.	Methods 2.4	Index classification was binary: physician-documented clinical recognition present vs absent before testing; no quantitative cutoff.
12b	Comparator	Define and justify positivity/categorical cutoffs for the comparator.	Methods 2.7–2.8; Discussion	FEV_1/FVC <80% plus FEV_1 increase ≥12% after bronchodilator; limitations of fixed threshold and newer ATS/ERS approaches are acknowledged.
13a	Blinding	State whether comparator interpretation was done without knowledge of index test.	Methods 2.3–2.4	Prior clinical recognition was documented before interpretation of the respiratory assessment, reducing incorporation from subsequent test results.
13b	Blinding	State whether index-test interpretation was done without knowledge of comparator.	Methods 2.3–2.4	Yes by temporal definition: clinical recognition was recorded before standardized spirometric classification.
14	Analysis	Describe methods for estimating/comparing accuracy measures.	Methods 2.11	Yes – sensitivity, specificity, predictive values, likelihood ratios, Cohen κ, 95% CI methods, and restricted two-gate interpretation are specified.
15	Analysis	Explain handling of indeterminate/unclassifiable test results.	Methods 2.8–2.12; Table S1	Yes – 72 participants retained as unclassified, excluded from primary classified-only comparison, included in two extreme-case sensitivity analyses.
16	Analysis	Explain handling of missing data.	Methods 2.12	Yes – no imputation; complete-case primary analysis; inability to separate technical failure vs missing post-bronchodilator testing vs extraction gaps is disclosed.
17	Analysis	Describe analyses of variability, distinguishing prespecified from exploratory.	Methods 2.11; Results 3.4	Exploratory comparisons are labeled unadjusted and post hoc; no multiplicity adjustment or multivariable model was used.
18	Sample size	State intended sample size and how it was determined.	Methods 2.2; Results 3.1	No prespecified diagnostic-accuracy sample-size calculation is reported; the secondary analysis used 140 available routine-care records. Eligible denominator was unavailable.
19	Participant flow	Provide a participant flow diagram.	Main Figure 1; Supplementary Figure S1	Yes.
20	Baseline characteristics	Report baseline demographic and clinical characteristics.	Table 1	Yes.
21a	Disease spectrum	Report severity distribution in participants with target condition.	Not strictly applicable	No definitive target disease/reference-standard diagnosis was assigned; reversible obstruction is an operational spirometric phenotype.
21b	Alternative diagnoses	Report alternative diagnoses in participants without target condition.	Not strictly applicable	The normal-spirometry comparator is not a disease-negative reference group; alternative diagnoses were not systematically adjudicated.
22	Timing/interventions	Report time interval and interventions between index and comparator tests.	Methods 2.3–2.7	Clinical recognition was documented before standardized testing; spirometry and bronchodilator reversibility were performed within the respiratory assessment. The index is a pre-existing clinical record status rather than a same-day test.
23	Cross-tabulation	Provide cross-tabulation of index results by comparator results.	Table 2; Table S2	Yes – full 3×2 table: reversible obstruction, normal spirometry, unclassified × prior suspicion yes/no.
24	Accuracy estimates	Report accuracy estimates with precision.	Abstract; Results 3.2–3.3; Table 3	Yes – sensitivity, specificity, PPV, NPV,

Item	Section/topic	STARD 2015 requirement (paraphrased)	Location in manuscript	Completion/applicability comment
				LR+/LR−, κ and 95% CIs; primary table reports core classification measures with CIs.
25	Adverse events	Report adverse events from index/comparator testing.	Ethics/Methods	No research-specific examination or intervention was added by this secondary analysis. The manuscript does not report test-related adverse-event data.
26	Discussion	Discuss limitations including bias, statistical uncertainty, and generalisability.	Discussion, pp. 16–17	Yes – extensive limitations include post hoc comparator, two-gate design, 51.4% unclassified, missing denominator, fixed threshold, unavailable covariates/tests, and imprecision.
27	Discussion	Discuss implications for practice, including intended use/clinical role.	Discussion; Conclusions	Yes – routine symptoms should trigger, not replace, objective respiratory assessment; integration of pediatric SCD and pulmonary care is advocated.
28	Other information	Provide registration number and registry name.	Not reported	Not registered as a diagnostic-accuracy study; this was a secondary analysis of routine-care data.
29	Other information	State where the full protocol can be accessed.	Methods 2.13 / IRB statement	The broader DREPASTHME framework is identified (version 1.0, 22 March 2024), but no public protocol-access location is reported.
30	Other information	Report funding/support and role of funders.	Funding statement	Yes – no specific grant from public, commercial, or not-for-profit sectors.

Note: STARD 2015 contains 30 essential reporting items plus a participant flow diagram. This completed mapping is supplied because the manuscript reports sensitivity, specificity, predictive values, likelihood ratios, and Cohen κ, while explicitly acknowledging that the operational spirometric comparator is not an independent diagnostic reference standard. Locations refer to the JCM final proof and may shift slightly during production.
