# Supplementary material for: Discordance Between Clinical Suspicion and Spirometry-Defined Reversible Airflow Obstruction in Children with Sickle Cell Disease in French Guiana
Source: J Clin Med. 2026 Aug 17;15(16):6342. doi: 10.3390/jcm15166342 (PMC13513272; doi:10.3390/jcm15166342)
Supplement: Supplementary file 1 [file jcm-15-06342-s001.zip › JCM_4488223_Supplementary_Materials_FigureS1_TablesS1-S3.pdf]

Journal of Clinical Medicine (JCM) – Supplementary Material

Discordance Between Clinical Suspicion and Spirometry-Defined Reversible Airflow Obstruction in Children with Sickle Cell Disease in French Guiana

Manuscript ID: jcm-4488223

Gabriel Bafunyembaka, Estiverne Evens, Nadia Nathan, Arriel Makembi, Narcisse Elenga

Complete Supplementary Materials: Figure S1 and Tables S1–S3

Figure S1. Operational respiratory-classification algorithm.

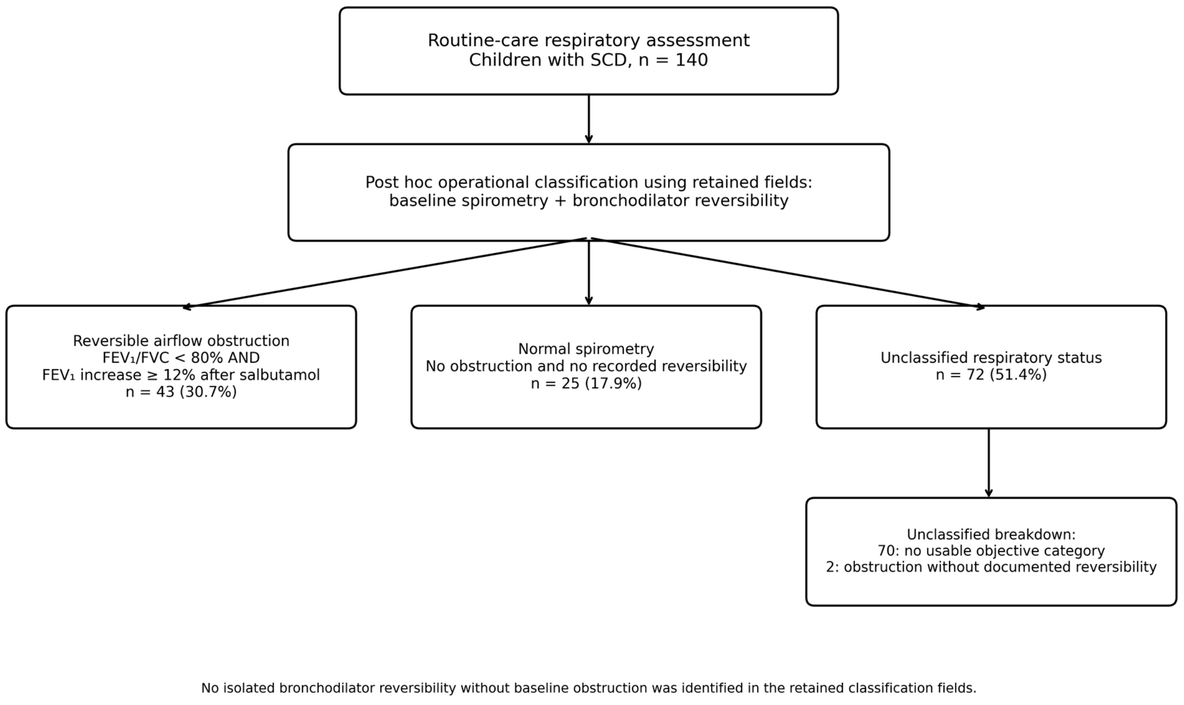

Note: Reversible obstruction required FEV_1/FVC <80% and an FEV_1 increase ≥12% from baseline 15 min after salbutamol 400 µg delivered through a spacer. The operational comparator was developed post hoc during peer-review revision and is not a diagnostic gold standard for asthma.

Table S1. Reasons for unclassified respiratory status among 72 children with sickle cell disease.

Reason for unclassified respiratory status	Unclassified, n = 72
No usable objective respiratory category in the retained extract	70 (97.2)
Airflow obstruction without documented bronchodilator reversibility	2 (2.8)
Total	72 (100.0)

Note: Data are n (%). The retained extract did not allow the 70 records without a usable objective category to be separated into technically unacceptable maneuvers, absent post-bronchodilator testing, or incomplete data capture. Unclassified participants were excluded from the primary classified-only comparison and considered in extreme-case sensitivity analyses.

Table S2. Prior clinical suspicion by mutually exclusive respiratory classification.

Prior clinical suspicion	Reversible obstruction	Normal spirometry	Unclassified	Total
Yes	10	3	22	35
No	33	22	50	105
Total	43	25	72	140

Note: Clinical suspicion was recorded before standardized respiratory testing. Among 43 children with reversible obstruction, 33 (76.7%) had no prior clinical suspicion.

Table S3. Performance of prior clinical suspicion in the primary and sensitivity analyses.

Analysis	Sensitivity, % (95% CI)	Specificity, % (95% CI)	PPV, % (95% CI)	NPV, % (95% CI)	Cohen κ (95% CI)
Primary: unclassified excluded	23.3 (11.8–38.6)	88.0 (68.8–97.5)	76.9 (46.2–95.0)	40.0 (27.0–54.1)	0.090 (−0.059–0.239)
Sensitivity: unclassified negative	23.3 (11.8–38.6)	74.2 (64.3–82.6)	28.6 (14.6–46.3)	68.6 (58.8–77.3)	−0.027 (−0.183–0.139)
Sensitivity: unclassified positive	27.8 (19.9–37.0)	88.0 (68.8–97.5)	91.4 (76.9–98.2)	21.0 (13.6–30.0)	0.070 (−0.003–0.145)

Note: PPV, positive predictive value; NPV, negative predictive value; CI, confidence interval. The primary analysis deliberately compared 43 children with reversible obstruction with 25 children with normal spirometry. Sensitivity analyses treated all 72 unclassified participants as outcome-negative or outcome-positive. These extreme-case analyses illustrate sensitivity to missing classification and should not be interpreted as general screening-performance estimates.
